# Prediction of Biochar Adsorption of Uranium in Wastewater and Inversion of Key Influencing Parameters Based on Ensemble Learning

**DOI:** 10.3390/toxics12100698

**Published:** 2024-09-26

**Authors:** Zening Qu, Wei Wang, Yan He

**Affiliations:** College of Mechanical and Electrical Engineering, Northeast Forestry University, Harbin 150040, China; zeningqu@nefu.edu.cn (Z.Q.); holy_he@nefu.edu.cn (Y.H.)

**Keywords:** wastewater treatment, uranium adsorption, biochar, ensemble learning, parameter inversion

## Abstract

With the rapid development of industrialization, the problem of heavy metal wastewater treatment has become increasingly serious, posing a serious threat to the environment and human health. Biochar shows great potential for application in the field of wastewater treatment; however, biochars prepared from different biomass sources and experimental conditions have different physicochemical properties, resulting in differences in their adsorption capacity for uranium, which limits their wide application in wastewater treatment. Therefore, there is an urgent need to deeply explore and optimize the key parameter settings of biochar to significantly improve its adsorption capacity. This paper combines the nonlinear mapping capability of SCN and the ensemble learning advantage of the Adaboost algorithm based on existing experimental data on wastewater treatment. The accuracy of the model is evaluated by metrics such as coefficient of determination (R^2^) and error rate. It was found that the Adaboost–SCN model showed significant advantages in terms of prediction accuracy, precision, model stability and generalization ability compared to the SCN model alone. In order to further improve the performance of the model, this paper combined Adaboost–SCN with maximum information coefficient (MIC), random forest (RF) and energy valley optimizer (EVO) feature selection methods to construct three models, namely, MIC-Adaboost–SCN, RF-Adaboost–SCN and EVO-Adaboost–SCN. The results show that the prediction model with added feature selection is significantly better than the Adaboost–SCN model without feature selection in each evaluation index, and EVO has the most significant effect on feature selection. Finally, the correlation between biochar adsorption properties and production parameters was discussed through the inversion study of key parameters, and optimal parameter intervals were proposed to improve the adsorption properties. Providing strong support for the wide application of biochar in the field of wastewater treatment helps to solve the urgent environmental problem of heavy metal wastewater treatment.

## 1. Introduction

With the growing global demand for clean energy, the importance of nuclear power as a low-carbon and highly efficient form of energy is becoming increasingly important [1]. However, the development of nuclear power is faced with the challenge of nuclear waste disposal, especially uranium-containing wastewater, and stringent and effective measures need to be taken to ensure safe disposal [2]. On the one hand, the chemical and radiological hazards of uranium are significant due to its long half-life and high toxicity [3], posing a threat to human health and natural ecology that cannot be ignored. On the other hand, as an indispensable component of nuclear fuel, uranium resources are precious and reserves are limited, making the mining and recycling of uranium more important [4]. Currently, many technologies are used to treat uranium-containing wastewater, such as membrane separation [5], solvent extraction [6], chemical precipitation [7], ion exchange [8] and adsorption [9]. Regarding economic and practical advantages, adsorption technology has gradually attracted people’s attention to treating uranium-containing wastewater due to its convenient operation, low operating cost, high efficiency and environmental protection [10].

Adsorbent materials such as minerals, which are widely used today, generally face the problems of low renewability, difficult biodegradation and the possibility of secondary pollution [11]. Biochar, as a high carbon content and porous adsorption medium, has shown great potential for application in the field of wastewater purification and environmental pollution remediation due to its unique structural properties, wide accessibility, ecological friendliness, recyclability and cost-effectiveness [12]. Biochar is rich in negatively charged functional groups that bind to heavy metal ions in contaminated water to form complexes, and these complexes can fix the heavy metal ions on the surface of the biochar, thus providing a new and effective method for the treatment of heavy metal contaminated water [13]. However, different biomass sources and experimental conditions can produce biochar with different physicochemical properties, leading to different adsorption capacities for U (VI) [14]. Many biochars perform poorly in adsorption efficiency, limiting their wide application in wastewater treatment. Given this, there is an urgent need to explore in-depth and optimize the setting of key parameters of biochar to significantly enhance its adsorption capacity.

Chen et al. [15] studied the adsorption of U (VI) on hydrothermal biochar, and the maximum adsorption capacity was 388.81 mg/g at 298 K. They found that adsorption capacity highly depends on solution pH, surface functional groups and contact time. Yakout [16] investigated the adsorption of U (VI) on rice straw biochar and showed that the adsorption is governed by the biochar’s structural features and surface functional groups, which may be affected by the pH of the solution. Yang et al. [17] combined hydrothermal and pyrolysis processes to prepare magnesium-containing lotus pod-derived biochar (MgO-HBC), which enhanced the adsorption of uranium by increasing the surface area, thermal stability and active sites of the biochar. Conventional experimental studies of uranium adsorption are often costly in terms of human, material and financial resources due to their complexity and safety risks.

With the continuous development of artificial intelligence, some scholars, based on the currently large amount of experimental data accumulated, propose to study how to improve the adsorption capacity of biochar to heavy metal uranium through machine learning (ML) and explore the correlation between the key parameters of biochar and adsorption performance, in order to reduce the use of uranium and avoid the harm of uranium radiation to the human body in actual experiments. Guo et al. [18] established an artificial neural network ANN model with a total of eight input parameters, including initial metal concentration C_0_ (μg/L) and solution pH, to predict the adsorption capacity of heavy metals, with a high correlation coefficient (R) of 0.926~0.994. Zhu et al. [19] used artificial neural networks (ANN) and random forests (RFs) to model the adsorption efficiency of heavy metals on biochar. The prediction accuracy of RF is better than that of the ANN model. In order to further improve the efficiency and effect of machine learning, Wang et al. [20] proposed the stochastic configuration network (SCN). Compared with deep neural networks (DNN) and convolutional neural networks (CNN), SCN not only ensures high prediction accuracy, but also significantly reduces computing costs, is more suitable for processing large-scale data sets and can quickly output prediction results. In addition, SCN’s strong generalization performance ensures good adaptability and stability of data under different experimental conditions.

Limited by the very long time of machine learning using optimization algorithms, many scholars have used integrated learning with more stable performance for prediction. Liu et al. [21] proposed four different hybrid methods, including GD-ALR-BP, GDM-ALR-BP, CG-BP-FR and BFGS, for wind speed prediction based on the Adaboost algorithm and multilayer perceptron neural networks. Yan et al. [22] proposed an integrated model based on the rolling decomposition method and deep learning algorithms for predicting NH_3_-N concentration in wastewater. However, wastewater treatment of heavy metals involves a complex environment, and numerous factors affect biochar’s adsorption capacity on uranium, such as the specific surface area (SA, m^2^/g) of the biochar, the mass percentage of total carbon and the temperature. These factors not only increase the difficulty of modeling, but also features with poor correlation will affect machine learning. In order to make the recognition effect of machine learning more satisfactory, researchers have proposed the feature selection method. Guo et al. [23] proposed a hybrid method of random forest regression and maximum information coefficient (RFR-MIC), and it was found that the prediction model with the addition of feature selection had higher prediction accuracy. Therefore, in this paper, in constructing the model, the three methods of maximum information coefficient (MIC), random forest (RF) and energy valley optimizer (EVO) are selected for feature selection, thus helping the machine learning model to be trained quickly, and also improving the accuracy of the model and reducing overfitting [24].

In view of the high cost, long period and uncertainty of traditional biochar parameter determination methods, the inversion method was innovatively introduced and applied in this paper [25]. This method not only reduces the research cost and time consumption significantly, but also obtains the optimal parameter value efficiently and accurately by the way of backward calculation. Mu et al. [26] proposed a reverse analysis method based on field test data, which can predict the three-dimensional displacement of soil caused by support excavation. Therefore, the inversion method is given priority in this paper to solve for the optimal parameter values of biochar. The limitation of traditional methods is overcome effectively, and a new way is opened up for the rapid and accurate determination of biochar parameters.

In order to deal with the increasingly serious water pollution problem, the prediction model of uranium adsorption by biochar is more stable and the training speed is faster. This paper adopts the nonlinear mapping capability of SCN and the ensemble learning advantage of the Adaboost algorithm. The maximum information coefficient (MIC), random forest (RF) and energy valley optimizer (EVO) feature selection methods are combined to construct five kinds of prediction models of uranium adsorption capacity: SCN, Adaboost–SCN, MIC-Adaboost–SCN, RF-Adaboost–SCN, EVO-Adaboost–SCN. Based on a large amount of experimental data, this study predicted the adsorption capacity of different biochar materials for uranium and inverted the key parameters to provide a reference for biochar efficient treatment of uranium pollution.

## 2. Materials and Methods

### 2.1. Data Collection and Preprocessing

This paper collected 546 groups of experimental data on uranium adsorption by biochar from references [27,28,29,30,31,32,33,34]. The physical and chemical properties of biochar and experimental conditions were used as input parameters for the model. The physical properties of biochar include specific surface area (SA, m^2^/g), average pore size (D, nm) and total pore volume (V, cm^3^/g). Chemical properties include the mass percentage of total carbon (C, %), the molar ratio of oxygen to carbon (O/C) and the molar ratio of oxygen to nitrogen [(O + N)/C]. The experimental conditions include pH, temperature (T, K), initial concentration of uranium (C_0_, mg/L) and solid–liquid ratio (SLR, g/L). The adsorption capacity of uranium on biochar (AC, mg/g) is taken as the output parameter [35].

It is shown in Table 1 that 546 sets of experimental data cover a variety of experimental conditions and the physical and chemical properties of biochar, and the numerical ranges and units of different features are quite different, which will cause the model to attribute too much weight to some features during training while ignoring other important features with small numerical values, thus affecting the accuracy of the model. In order to solve this problem, the min–max normalization method was adopted to convert data with different features into the specified range [0, 1] through linear transformation of the original data, in order to eliminate the dimensional differences between different features and make all features equal in model training [36]. The specific formula is shown as (1). The normalization reduces the influence of data range difference on gradient calculation and enables the model to converge to the optimal solution faster during training. This not only saves training time, but also reduces the consumption of computing resources. The normalized data make it easier for the model to learn the relative importance between the features, thus improving the prediction accuracy and stability of the model.

(1) $$X^{*}=\frac{X_{i}-X_{min}}{X_{max}-X_{min}}$$

In Formula (1), X_i_ is the raw data, such as the physical and chemical properties and experimental conditions of biochar to be normalized; X^*^ is the normalized value; X_max_ is the maximum value of the original data. X_min_ is the minimum value of the original data.

### 2.2. Ensemble Learning Theory

#### 2.2.1. SCN

**Figure 1:**
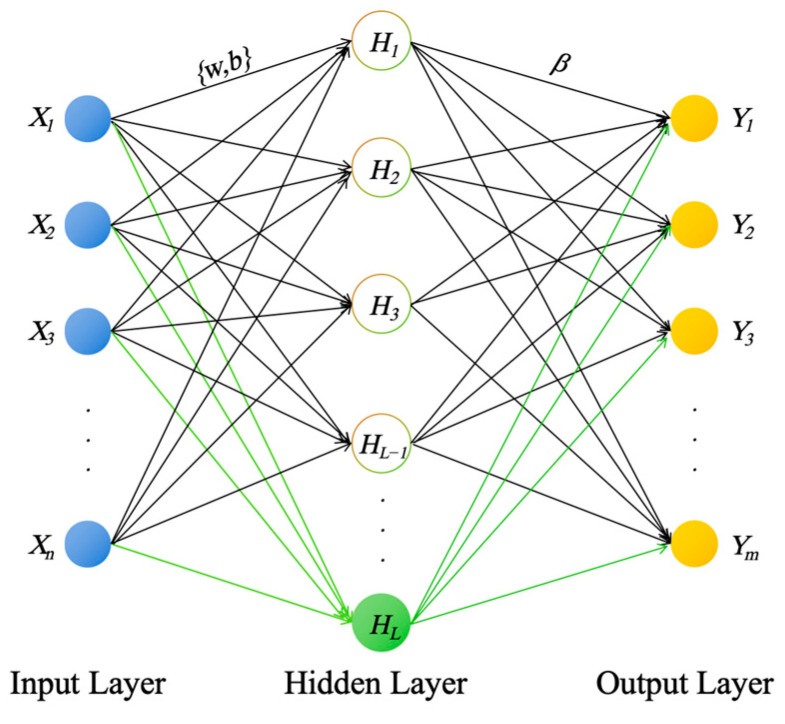
Structure of SCN network.

The SCN is widely used as a random network to process data regression and classification [37]. SCN is a random weight neural network, similar to a feedforward neural network, including input, hidden and output layers. The structure of SCN network is shown in Figure 1. X_1_, X_2_, ⋯ X_n_ is the input sample, Y_1_, Y_2_, …, Y_m_ is the output variable, the input weight w and bias b are generated by the input layer and sent to the hidden layer, and the hidden layer sends the output weight β to the output layer.

During the whole construction process, the number of nodes in the input layer and the number of nodes in the output layer correspond to the dimensions of the training input and output features, respectively. In contrast, the number of nodes in the hidden layer gradually increases with the construction process [38]. The input weights and biases of hidden layer nodes are affected by λ and r. After adding hidden layer nodes, a candidate layer is constructed to determine the weight and bias of the new node. The initialization of the weight and bias of the candidate layer nodes depends on the scale factor λ, and the calculation of inequality constraints in the supervision mechanism depends on the regularization parameter r. The optimal weights and deviations of the candidate layer nodes are selected using the special monitoring mechanism to calculate inequality constraints and then assigned to the new nodes. Finally, the weight of the output layer is calculated using the least squares method according to the output node output and output vector of the hidden layer.

SCN and other neural networks differ in their unique training modes. It abandons the traditional iterative method of updating network parameters. It adopts the mode of increasing hidden layer nodes under constraints, which makes the network size flexible and controllable and greatly reduces the consumption of computational resources.

#### 2.2.2. Adaboost Ensemble Framework

**Figure 2:**
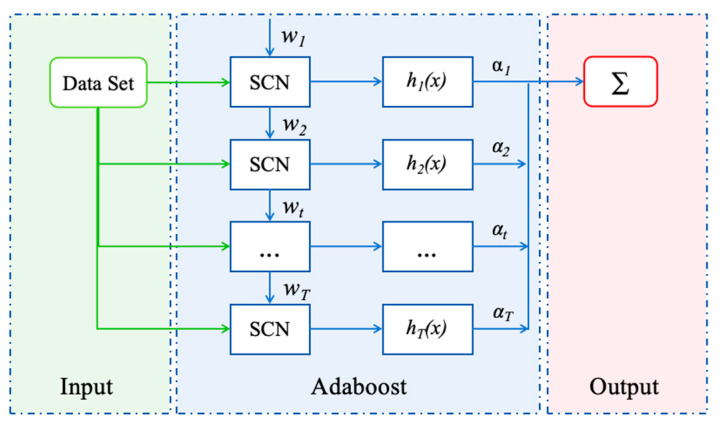
Training process of Adaboost–SCN.

Adaboost is a typical boosting algorithm, an ensemble learning algorithm. The flow chart of the Adaboost–SCN algorithm is shown in Figure 2. Its operating principle is similar to the human learning process, and each learning will further adjust its weight for some relatively high error samples. The strong learner synthesized by the Adaboost method retains the advantages of a single weak learner and weakens its disadvantages [39].

The algorithm first assigns an initial weight matrix w_t_ to the sample set and trains the first sub-learner h_t_(x), then calculates the weight of the sub-learner and the new weight matrix of the sample set according to the training error and starts a new round of training [40]. As can be seen from the flow chart in Figure 2, in the iterative step, the strong learner generated in the previous iteration will be used in the next iteration, thus generating a new strong learner. The formula is as follows:

(2) $$H_{t}(x)=H_{t-1}(x)+\alpha_{t}h_{t}(x)$$

In Formula (2), H_t_(x) is the strong learner obtained by the t iteration, H_T−1_ (x) is the strong learner obtained by the t − 1 iteration, H_t_(x) is the sub-learner, t is the number of iterations and α_t_ is the weight matrix of the sub-learner. When the maximum number of iterations T is reached, the strong learner is synthesized according to the coefficient of the weak learner, as shown in Formula (3).

(3) $$H(x)=\sum_{t=1}^{T}\alpha_{t}h_{t}(x)$$

In Formula (3), H(x) is a strong learner obtained by a linear combination of sub-learners, and T is the maximum number of iterations.

### 2.3. Feature Selection Method

Feature selection is an important problem in feature engineering. Its goal is to select the features that are important to the model, in order to reduce the number of features, improve the accuracy of the model and reduce the running time. According to different selection strategies, it can be divided into filter, embedded and wrapper [41].

#### 2.3.1. Filter—Maximum Information Coefficient (MIC)

The maximum information coefficient (MIC) belongs to the filtering method, which is an algorithm for correlation analysis based on mutual information. It adopts the method of grid division, which has better universality, fairness and symmetry [42]. MIC is used to measure the degree of correlation between two variables; the MIC value ranges from [0, 1]; when the MIC value between two variables is larger, the correlation is stronger, and vice versa, the smaller the MIC value is, the smaller the correlation of the two variables is. The basic principle of MIC uses the concept of mutual information, and the formula of mutual information and MIC is shown in Equation (4).

(4) $$I(x;y)=\int p(x,y){log}_{2}\frac{p(x,y)}{p(x)p(y)}dxdy$$

In Formula (4), x and y are two vectors, p(x,y) is the joint probability density of vectors x and y, and p(x) and p(y) are the marginal probability densities of vectors x and y, respectively. A two-dimensional rectangular coordinate system is established, the scatter plot composed of vectors x and y is divided into a certain number of grids, and the scatter distribution of each grid is checked to obtain the joint probability [43]. The MIC calculation formula is shown in Equation (5) as follows:

(5) $$MIC(x;y)=\underset{a\times b<B(N)}{\max}\frac{I(x;y)}{{log}_{2}min(a,b)}$$

where N is the number of samples and B(N) is the function of samples, indicating that the total number of small units a × b of the grid is less than B(N), and B(N) is generally set as approximately the power of 0.6 of the total amount of data N, that is, B(N) = N^0.6^.

#### 2.3.2. Embedded—Random Forest (RF)

The embedded method simultaneously performs feature selection while training machine learning algorithms. The feature selection process is embedded into the model construction process, and some model features are used for automatic feature selection. In the field of feature elimination, random forest (RF), as a method that fuses different decision trees based on integration technology, can effectively process high-dimensional feature input samples with redundant data, evaluate the importance of each feature of the data in classification problems and realize importance ranking [44].

Random forests can calculate the importance of features and rank them. There are three common methods for estimating importance: frequency of statistical features such as segmentation feature, gini index and OOB data calculation error value. We selected OOB data to calculate the error value and sort the features. The steps are as follows: First, n sets of OOB data are used to calculate the error value of each decision tree, denoted as Err_OOB1_, Err_OOB2_, … Err_OOBn_. Then, noise interference was randomly added to feature i of all OOB samples, and we ensured that other features remain unchanged, and the error values were recalculated, denoted as Err_i1_, Err_i2_, … Err_in_. The formula for calculating the importance of features is as follows:

(6) $${Import}_{x}=\frac{1}{n}\sum_{m=1}^{n}\left({Err}_{im}-{Err}_{OOBm}\right)$$

#### 2.3.3. Wrapper—Energy Valley Optimizer (EVO)

The wrapper method is a feature selection method that combines a feature selection process with a machine learning algorithm. It evaluates the effectiveness of different feature subsets by training and testing a specific machine learning algorithm to select the optimal feature subset. The wrapper method relies on the learning model’s classification accuracy or prediction accuracy as an evaluation criterion for feature selection.

The energy valley optimizer (EVO) is a novel meta-heuristic algorithm inspired by physical principles regarding stability and different particle decay patterns [45]. In the universe, most particles are thought to be unstable, with each particle moving toward the bottom of the energy valley to increase its level of stability [46]. The position of the particle is between 0 and 1. If the feature value represented by the particle is less than 0.5, it means that the feature is selected. If the value is greater than 0.5, the feature is not selected. The position updates for the energy valley optimizer are as follows. The first step is initialization, assuming that the search space is the specified part, and assuming that the candidate solution (X_i_) is a particle with different stability levels in the search space. In the second step, each particle is evaluated by an objective function to determine the particle’s enrichment boundary (EB) and neutron enrichment level (NEL), which are used to account for differences between neutron-rich and neutron-poor particles. The specific formula is expressed as follows:

(7) $$EB=\frac{\sum_{i=1}^{n}{NEL}_{i}}{n},i=1,2,\ldots ,n$$

where NEL_i_ is the neutron enrichment level of particle i, and EB is the enrichment boundary of particles in the universe.

The third step is to evaluate the stability level of the particle according to the objective function, the specific formula is expressed as follows:

(8) $${SL}_{i}=\frac{{NEL}_{i}-BS}{WS-BS},i=1,2,\ldots ,n$$

where SL_i_ is the stability level of the ith particle, BS and WS are the most and least stable particles in the universe, respectively, and the minimum and maximum of the objective function determine their stability levels.

According to the stability of the particles, three decay processes (α, β, γ) are used corresponding to three position renewal processes. In the main search cycle of the EVO, if the neutron enrichment level of a particle is above the enrichment boundary (NEL_i_ > EB), it is assumed that the particle has a higher neutron to proton ratio. Therefore, it is correct to adopt three decay processes (α, β, γ). To simulate the stability boundary (SB) in the universe, a random number is generated within the shown interval [0, 1]. α and γ decays are considered to have occurred if the stability level of the particle is above the stability limit (SL_i_ > SB), since both decays can occur in heavier particles with higher stability levels. 

Take the α decay process, for example. According to the physical principle of decay, the rays emitted by α favor the enhancement of the stability of the reaction products. This process serves as one of the EVO position updating mechanisms, thus generating new candidate solutions. Specifically, two random integers are generated: Alpha Index I and Alpha Index II. d is the dimension of the problem under consideration, and Alpha Index I represents the number of emitted rays with the value range [1, d]. Alpha Index II defines the Alpha ray as emitted within the range [1, Alpha Index I]. The emitted ray is the decision variable in the solution candidate and is replaced by either the alpha ray in the particle or the candidate ray (X_BS_) with the best stable level. The specific formula is expressed as follows:

(9) $$X_{i}^{New1}=X_{i}\left(X_{BS}\left(X_{i}^{j}\right)\right),\left\{\begin{array}{c} i=1,2,\ldots ,n.\\ j=Alpha\;Index\;II. \end{array}\right.$$

In Formula (7), $X_{i}^{New1}$ is the newly generated particle in the universe, X_i_ is the current position vector of the ith particle (candidate solution) in the universe, X_BS_ is the position vector of the particle with the best stable level and $X_{i}^{j}$ is the jth decision variable or emission ray.

### 2.4. Prediction Model of Uranium Adsorption by Biochar

**Figure 3:**
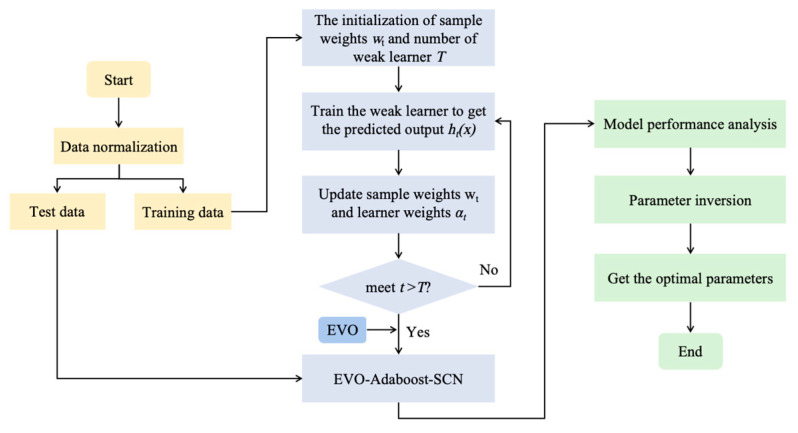
Implementation process of EVO-Adaboost–SCN.

In this research, five kinds of uranium adsorption capacity prediction models were established by MATLAB: SCN, Adaboost–SCN, MIC-Adaboost–SCN, RF-Adaboost–SCN and EVO-Adaboost–SCN. The implementation process of EVO-Adaboost–SCN is shown in Figure 3. Of the 546 sets of experimental data on uranium adsorption by biochar collected, 70% represent the training set of the model and 30% are the test set.

The specific settings of the model are as follows: the maximum number of hidden nodes is 40, the maximum number of candidate nodes is 100, the error limit is 0.01, the error threshold is 0.02 and six weak learners are set, that is, six weak learners score the strong learning period. Secondly, three feature selection methods, MIC, RF and EVO, were combined with the Adaboost–SCN model to construct the MIC-Adaboost–SCN, RF-Adaboost–SCN and EVO-Adaboost–SCN models. The performance of different models was compared, and inversion research was carried out based on the optimal model. Inversion was carried out with the maximum output value as the target, the number of iterations was set to 100, the interval of iteration was based on the upper and lower boundaries of the original data, the population size was 30 and the optimal key parameter settings were obtained through inversion.

### 2.5. Validation of Model Accuracy and Reliability

We determined the accuracy of the model based on the BP neural network by determining the coefficient (R^2^) and error rate. Error rates include mean absolute error (MAE), mean square error (MSE), root mean square difference (RMSE) and mean absolute percentage error (MAPE) [47].

(10) $$R^{2}=1-\frac{\sum_{i=1}^{N}{\left(y_{i}-\hat{y_{i}}\right)}^{2}}{\sum_{i=1}^{N}{\left(y_{i}-\bar{y}\right)}^{2}}$$

(11) $$MAE=\frac{1}{N}\sum_{i=1}^{N}\left|y_{i}-\hat{y_{i}}\right|$$

(12) $$MSE=\frac{1}{N}\sum_{i=1}^{N}{\left(y_{i}-\hat{y_{i}}\right)}^{2}$$

(13) $$RMSE=\sqrt{\frac{1}{N}\sum_{i=1}^{N}\left|y_{i}-\hat{y_{i}}\right|}$$

(14) $$MAPE=\frac{1}{N}\sum_{i=1}^{N}\left|\frac{y_{i}-\hat{y_{i}}}{y_{i}}\right|\times 100\%$$

In the above formula, y_1_, y_2_, …, y_n_ are the true values of uranium adsorbed by biochar; $\bar{y}$ is the average of all true values; ${\hat{y}}_{1},{\hat{y}}_{2},\ldots {\hat{,y}}_{n}$ are the predicted values of uranium adsorption by biochar; and $y_{i}-\hat{y_{i}}$ is the residual of the i sample, representing the difference between the predicted value of biochar adsorbed uranium and the true value, which can better reflect the actual situation of the predicted value error.

## 3. Results and Discussion

### 3.1. Comparative Analysis of Prediction Models with or without Integrated Framework

Compared with the single SCN model, Adaboost–SCN showed significant advantages in prediction accuracy, accuracy, model stability and generalization ability.

As shown in Table 2, the MAE of Adaboost–SCN is 8.7600, which is lower compared to the 10.1287 of SCN, indicating that the Adaboost-integrated model has a smaller average absolute difference between predicted and actual values and a higher prediction accuracy. The MSE, RMSE and MAPE of Adaboost–SCN are lower than their counterparts in SCN. This further demonstrates the superiority of the integrated model in reducing the prediction error. The R^2^ value of Adaboost–SCN is 0.9768, which is higher than the 0.9673 of SCN, indicating that the integrated model has a better fit between the predicted and actual values.

While individual SCN models may have been optimized to avoid overfitting, Adaboost’s integration mechanism further diminishes the risk of overfitting a single model by fusing multiple weaker learners together to construct a single powerful learner, endowing the overall model with greater perturbation resistance and robustness. Although a certain increase in computational cost accompanies these advantages, Adaboost’s integration approach is undoubtedly an efficient and worthwhile solution for pursuing superior predictive performance.

### 3.2. Comparative Analysis of Prediction Models with or without Feature Selection

The three models constructed by the three feature selection methods selected in this paper, MIC, RF and EVO, were compared with the evaluation indicators of the models without feature selection, and the comparison results are shown in Table 2.

**Figure 4:**
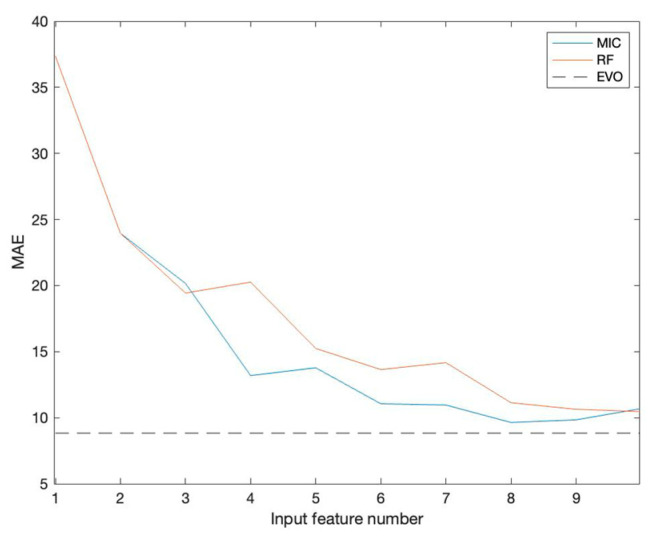
The variations in the absolute error (MAE) as the number of input features increases.

According to the values in Table 3, the prediction model with added feature selection is significantly better than the model without feature selection in each evaluation index. The average absolute error (MAE) is taken as the core fitness function for specific analysis. Features are introduced from high to low according to their importance assessment results. Figure 4 shows the variations in mean absolute error (MAE) as input features increase.

As the number of input features increases, the MAE of MIC and RF gradually decreases. An increase in the number of input features can enrich the dimensionality and information content of the data, thus helping the model to capture more details about the predicted target. On the contrary, when there are fewer input features, the information content of the data is limited, which may lead to less comprehensive information on which the model bases its prediction and thus increase the uncertainty of the prediction. In addition, a small number of features may also make the model overly dependent on a particular feature, resulting in biased prediction results.

When the number of input features reaches 8, the MIC algorithm’s performance becomes optimal. The RF algorithm shows the best performance when the number of features is increased to 10. However, neither MIC nor RF is as effective as the EVO algorithm in feature selection. This comparison highlights the superiority of the EVO algorithm in feature selection, which can more effectively screen out the feature combination that contributes the most to model prediction, thus further reducing MAE and improving prediction accuracy.

**Figure 5:**
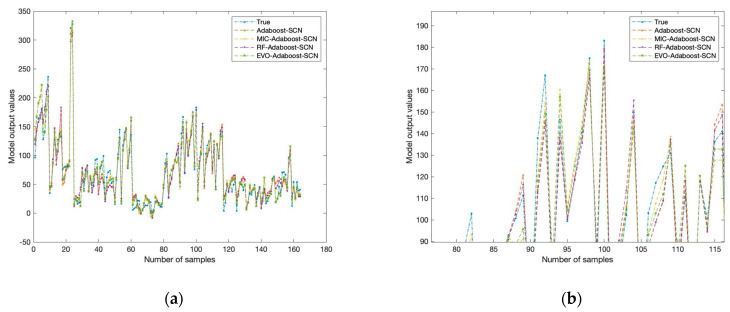
Test set fitting results for predicting uranium adsorption capacity of biochar: (**a**) Complete fitting results; (**b**) partial fitting results.

The fitting results of the four model test sets after training are shown in Figure 5b and Table 3. As shown in Figure 5b and Table 3, EVO-Adaboost–SCN has more obvious advantages than the other models. The EVO-Adaboost–SCN model has the best performance on the RMSE index, indicating that the difference between the predicted value and the actual value is the smallest, and the prediction accuracy is the highest. This is mainly due to the advantages of evolutionary algorithm in feature selection, which can more effectively screen out the features that have a positive impact on the model prediction performance. The R^2^ of the EVO-Adaboost–SCN model is 0.9849, which is not only better than the Adaboost–SCN model without feature selection, but also better than the MIC-Adaboost–SCN and RF-Adaboost–SCN models. This indicates that the energy valley optimization algorithm (EVO) in the EVO-Adaboost–SCN model is likely to help the model find better solutions in the training process through its optimization ability, thus improving the fitting effect and performance of the model on the test set. This advantage is reflected in the model’s ability to capture patterns in the data more accurately, handle unseen data more consistently and generalize more.

For the optimal EVO-Adaboost–SCN model, a robustness analysis was performed to evaluate its stability and performance in the face of input data variations, noise and outliers. Firstly, the sensitivity analysis of the model was carried out, and the change of key input parameters within the range of ±10% was set to simulate the normal fluctuation range that may be encountered in actual operation. The analysis results show that the EVO-Adaboost–SCN model can maintain relatively stable prediction accuracy under these parameter changes. In addition, the Monte Carlo simulation method was used in this study to generate different input data sets through multiple random sampling and run the model to make predictions to evaluate the stability of the model under uncertain conditions. The simulation results show that the distribution of prediction results of the model is relatively concentrated, which further proves its robustness and provides strong support for the reliability and effectiveness of the model in practical applications.

### 3.3. The Difference in Feature Selection

**Figure 6:**
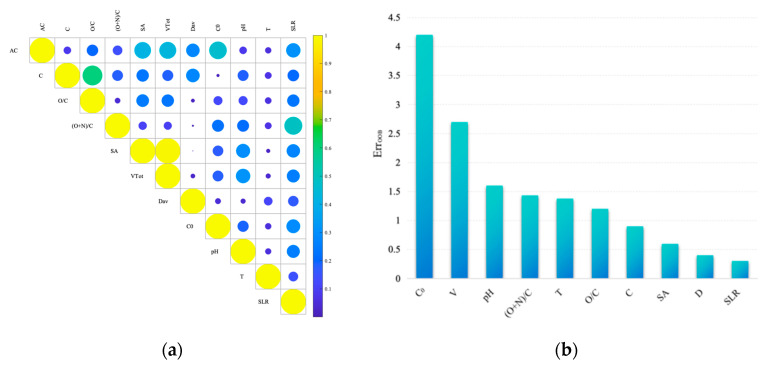
Feature selection results: (**a**) MIC; (**b**) RF.

We used the MIC, RF and EVO methods for feature selection. Figure 6a and Figure 6b show the feature selection results of MIC and RF, respectively.

The MIC is calculated based on the mutual information between variables, especially considering the nonlinear relationships between variables. The higher the maximum information coefficient, that is, the larger the area of the circle in the square, the stronger the correlation between the feature and the target variable. As can be seen from the results of Figure 6a, C_0_ and V have the strongest correlation with the target variable. In contrast, the correlation between C and T is weak. The maximum information coefficient of SA and V is 1, because V is proportional to SA. The ranking shown in Figure 6b is based on the importance of features in the random forest model, and RF also considers C_0_ and V as the two most important features. However, there are some differences between RF and MIC in ranking the remaining features, such as RF ranking pH before SA in MIC ranking, which indicates that different methods may focus on other data characteristics and model mechanisms when evaluating the importance of features.

Table 4 shows the feature selection results of the three methods. The results of MIC and RF are sorted according to the importance of features, while EVO only selects important features, and their order has nothing to do with the importance. According to Table 4, it can be seen that EVO filters the duplicate SA and V features and retains one of them. According to the selection of three features, C_0_ and V are relatively important. On the one hand, an increase in the initial concentration of uranium (C_0_) means that more uranium ions are available for adsorption, thus increasing the adsorption capacity. When the initial concentration of uranium (C_0_) is too large, the adsorption begins to slow down. On the other hand, a larger total pore volume (V) means more adsorption space and can provide more locations to accommodate uranium ions, allowing biochar to adsorb uranium more efficiently.

### 3.4. Key Parameter Inversion Results

**Figure 7:**
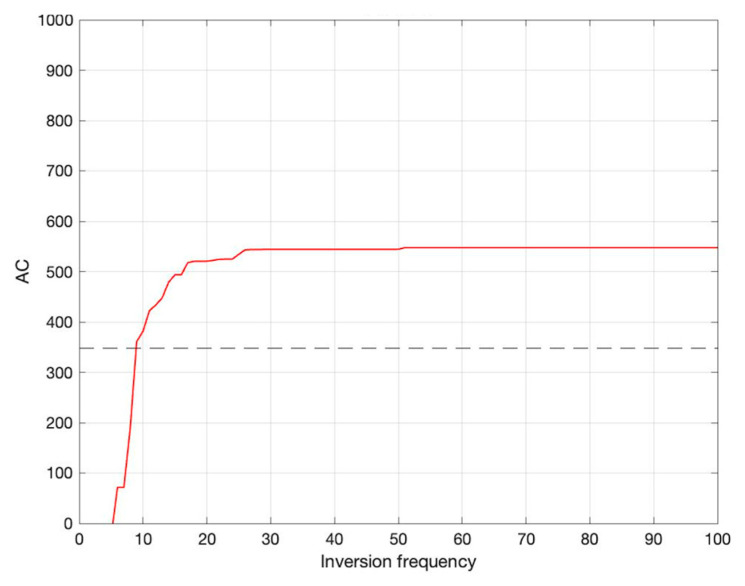
Inversion results.

Since the EVO-Adaboost–SCN model has the highest accuracy, this model was chosen for inversion research in this paper. The average absolute error (MAE) was used as the prediction performance index, the output value of the model was set as the main object of this inversion, and efforts were made to find the combination of input parameters that can produce the maximum output value. Since the result of each inversion is not repeatable, this study underwent 30 inversions, and Figure 7 shows the inversion results of one of them.

After 30 inversions, the optimal intervals of the key parameter settings were found. Specifically, C is [18.2, 22.43], O/C for [1.63, 2.14], (O + N)/C (1.48, 3), V is [0.29, 0.91], C_0_ is [19.8, 35.95], pH is [3.1, 8.2] and SLR is [0.1, 0.32]. The output value is up to 687.3. The results of inversion research are not only affected by the upper and lower boundaries of the iteration interval, but also by a variety of complex factors such as practical experimental conditions, time conditions, environmental conditions and specific application scenarios. These factors work together, so the inversion results may differ under different conditions.

Despite the preliminary results of this study on a new approach to wastewater treatment, there are limitations that could affect a full assessment. Assumptions made during the modeling process, while based on current scientific understanding and practical experience, may not fully consider all potential complicating factors. For example, the dynamic nature of wastewater composition with time limits the accuracy of prediction. Secondly, the constraints of available data are also a significant limitation of this study. Data sets may have deficiencies in coverage, temporal resolution and integrity, which can affect the model’s ability to learn comprehensive features, and data biases or outliers can interfere with model performance. In addition, the application of the results is limited by the size of the facility, operating conditions and other factors, and the applicability needs to be carefully evaluated. A comprehensive review and improvement of these limitations will promote the development of wastewater treatment and provide more accurate solutions.

### 3.5. Comparative Analysis of Model Performance

In a previous study, Qu et al. [48] explored the optimization effect of four different algorithms on BP neural networks, each of which improved the performance of the model to some extent, and Fick’s Law algorithm (FLA) had better search capability and convergence speed. Da et al. [35] applied four machine learning (ML) methods to make predictions and found that the model obtained with two hidden layer perceptron artificial neural networks performed best. Chen et al. [49] used six typical machine learning (ML) models to accurately predict the adsorption capacity of biochar and found that the CatBoost model showed the highest test R^2^ value and the lowest RMSE value, significantly superior to all other models. In contrast, the EVO algorithm adopted in this paper shows better efficiency and accuracy in searching for optimal parameters because of its unique evolutionary mechanism and powerful global search ability. In addition, the integrated model adopted in this paper not only integrates the advantages of a single model, but also effectively overcomes the possible limitations of a single BP model in complex prediction tasks.

In order to verify the superiority of the EVO-Adaboost–SCN model, the performance of this model was compared with that of a previously published model for predicting the adsorption of biochar materials. Specifically, linear regression (LR), support vector regression (SVR) and random forest (RF) models were included, and the comparison results are shown in Table 5.

According to Table 5, the EVO-Adaboost–SCN model performed exceptionally well, with an R^2^ of 0.9849, an almost perfect fit to the data, and an RMSE as low as 10.0432. The SVR model R^2^ is 0.92, with good predictive power but high RMSE (14.17). This may be due to the limitations of SVR model in dealing with complex data or nonlinear relationships, or its hyperparameters are not optimally adjusted. Although RF model is known for its strong generalization ability, the RMSE in this evaluation is high and the R^2^ is only 0.86, which may indicate that the RF model may have overfitting phenomenon, that is, the model performs well on the training data, but has poor generalization ability on the new data. The LR model performed the worst with an R^2^ of 0.37 and an RMSE of 32.36. This illustrates the limitations of linear regression models in dealing with this prediction task, as the task involves complex non-linear relationships or interactions that linear regression models cannot capture effectively. In summary, the EVO-Adaboost–SCN model has unique advantages and potential application prospects in the field of heavy metal adsorption prediction.

## 4. Conclusions

In this study, the Adaboost–SCN model was constructed using the idea of ensemble learning, and based on this model, combined with the feature selection method, three uranium adsorption prediction models for biochar were constructed: MIC-Adaboost–SCN, RF-Adaboost–SCN and EVO-Adaboost–SCN. These models not only make full use of the powerful integration capability of the Adaboost algorithm, but also accurately capture the key factors affecting the adsorption performance of biochar by the MIC, RF and EVO feature selection methods, in order to achieve a more accurate prediction of the uranium adsorption process.

1. The Adaboost–SCN model constructed in this paper is significantly superior to the single SCN model in terms of error rate, which fully validates the excellent performance of ensemble learning in reducing prediction errors and improving prediction accuracy. In order to accelerate the model training process and improve the prediction ability, three efficient feature selection methods were selected in this study: the maximum information coefficient (MIC), random forest (RF) and energy valley optimizer (EVO), which effectively helped the Adaboost–SCN model focus on key features. In particular, the Adaboost–SCN model combined with the EVO algorithm shows remarkable performance with an R^2^ value of 0.9849, which indicates that the predicted data are in good agreement with the experimental data;
2. The three feature selection methods, MIC, RF and EVO, have different results due to the differences in data characteristics and model mechanisms when evaluating the importance of features. Despite these differences, they all consider C_0_ and V relatively important features. In the process of uranium adsorption, with the increase in the initial concentration of uranium (C_0_), the number of uranium ions available for adsorption by biochar in the solution increased significantly, and the larger total pore volume (V) provided more abundant adsorption space for biochar, thus promoting the adsorption of uranium ions by biochar;
3. Through the inversion of key parameters, the correlation between biochar adsorption properties and production parameters was discussed, and the intervals of the optimal parameters were determined. These findings provide strong support for optimizing the biochar preparation process to improve its adsorption properties.

## Figures and Tables

**Table 1:** Partial data set on uranium adsorption by biochar.

	C	O/C	(O + N)/C	SA	V	D	C_0_	pH	T	SLR	Adsorption Capacity
1	54.55	0.297	0.61	275.30	0.212	3.14	11.99	5	293	0.10	96.32
2	37.60	1.080	1.18	363.19	0.259	8.65	118.02	4.5	298	0.25	332.57
3	77.14	0.048	0.1	446.31	0.235	9.49	100.01	6	298	0.25	66.46
4	50.55	0.068	5.37	363.19	0.259	8.65	15.34	6	298	0.20	73.54
…											
546	54.55	0.175	0.18	1298.00	0.919	8.65	47.60	7	298	0.40	171.39

**Table 2:** Comparison of evaluation indexes of prediction model with or without integrated framework.

	MAE	MSE	RMSE	MAPE	R^2^
SCN	10.1287	234.7781	15.3225	0.3067	0.9673
Adaboost–SCN	8.7600	154.8404	12.4435	0.2878	0.9768

**Table 3:** Comparison of evaluation indexes of a prediction model with or without an integrated framework.

	MAE	MSE	RMSE	MAPE	R^2^
SCN	10.1287	234.7781	15.3225	0.3067	0.9673
Adaboost–SCN	8.7600	154.8404	12.4435	0.2878	0.9768
MIC-Adaboost–SCN	8.3158	122.5791	11.0715	0.3160	0.9811
RF-Adaboost–SCN	9.0878	170.4979	13.0575	0.2946	0.9746
EVO-Adaboost–SCN	7.6100	100.8660	10.0432	0.2620	0.9849

**Table 4:** The results of three feature selection methods.

	1	2	3	4	5	6	7	8	9	10
MIC	C_0_	V	SA	SLR	D	O/C	(O + N)/C	pH	C	T
RF	C_0_	V	pH	(O + N)/C	T	O/C	C	SA	D	SLR
EVO	C	O/C	(O + N)/C	V	C_0_	pH	SLR			

**Table 5:** Comparative analysis of model performance.

	RMSE	R^2^
EVO-Adaboost–SCN	10.0432	0.9849
SVR [35]	14.17	0.92
LR [35]	32.36	0.37
RF [49]	44.995	0.86

## Data Availability

Data are available upon request from the corresponding author.
